# Predictors of Higher Quality of Systematic Reviews Addressing Nutrition and Cancer Prevention

**DOI:** 10.3390/ijerph19010506

**Published:** 2022-01-03

**Authors:** Dawid Storman, Magdalena Koperny, Joanna Zając, Maciej Polak, Paulina Weglarz, Justyna Bochenek-Cibor, Mateusz J. Swierz, Wojciech Staskiewicz, Magdalena Gorecka, Anna Skuza, Adam A. Wach, Klaudia Kaluzinska, Małgorzata M. Bała

**Affiliations:** 1 Chair of Epidemiology and Preventive Medicine, Department of Hygiene and Dietetics, Jagiellonian University Medical College, 31-034 Krakow, Poland; dawid.storman@doctoral.uj.edu.pl (D.S.); joanna.jankowska@uj.edu.pl (J.Z.); paulina.tobola@uj.edu.pl (P.W.); mateusz.swierz@doctoral.uj.edu.pl (M.J.S.); 2 Chair of Epidemiology and Preventive Medicine, Department of Epidemiology, Jagiellonian University Medical College, 31-034 Krakow, Poland; magdakoperny@gmail.com; 3 Department of Epidemiology and Population Studies, Institute of Public Health, Faculty of Health Sciences, Jagiellonian University Medical College, 31-034 Krakow, Poland; maciej.1.polak@uj.edu.pl; 4 Department of Radiation Oncology, St Lukas Hospital, 33-100 Tarnów, Poland; bochenekjm@gmail.com; 5 Students’ Scientific Research Group of Systematic Reviews, Jagiellonian University Medical College, 31-034 Krakow, Poland; staskiewicz.woj@gmail.com (W.S.); mgdgrck@gmail.com (M.G.); ania6495@gmail.com (A.S.); adamwach27@gmail.com (A.A.W.); ka.kaluzinska@gmail.com (K.K.)

**Keywords:** AMSTAR-2, ROBIS, quality, systematic review, meta-analysis, nutrition, cancer prevention

## Abstract

Systematic reviews/meta-analyses (SR/MAs) are considered a reliable source of information in healthcare. We aimed to explore the association of several characteristics of SR/MAs addressing nutrition in cancer prevention and their quality/risk of bias (using assessments from AMSTAR-2 and ROBIS tools). The analysis included 101 SR/MAs identified in a systematic survey. Associations of each specified characteristic (e.g., information about the protocol, publication year, reported use of GRADE, or other methods for assessing overall certainty of evidence) with the number of AMSTAR-2 not met (‘No’ responses) and the number of ROBIS items met (‘Probably Yes’ or “Yes’ responses) were examined. Poisson regression was used to identify predictors of the number of ‘No’ answers (indicating lower quality) for all AMSTAR-2 items and the number of ‘Yes’ or ‘Probably Yes’ answers (indicating higher quality/lower concern for bias) for all ROBIS items. Logistic regression was used to identify variables associated with at least one domain assessed as ‘low concern for bias’ in the ROBIS tool. In multivariable analysis, SR/MAs not reporting use of any quality/risk of bias assessment instrument for primary studies were associated with a higher number of ‘No’ answers for all AMSTAR-2 items (incidence rate ratio (IRR) 1.26, 95% confidence interval (CI) 1.09–1.45), and a lower number of ‘Yes’ or ‘Probably Yes’ answers for all ROBIS items (IRR 0.76, 95% CI 0.66–0.87). Providing information about the protocol and search for unpublished studies was associated with a lower number of ‘No’ answers (IRR 0.73, 95% CI 0.56–0.97 and IRR 0.75, 95% CI 0.59–0.95, respectively) and a higher number of ‘Yes’ or ‘Probably Yes’ answers (IRR 1.43, 95% CI 1.17–1.74 and IRR 1.28, 95% CI 1.07–1.52, respectively). Not using at least one quality/risk of bias assessment tool for primary studies within an SR/MA was associated with lower odds that a study would be assessed as ‘low concern for bias’ in at least one ROBIS domain (odds ratio 0.061, 95% CI 0.007–0.527). Adherence to methodological standards in the development of SR/MAs was associated with a higher overall quality of SR/MAs addressing nutrition for cancer prevention.

## 1. Introduction

The number of studies published as systematic reviews (SR) and meta-analyses (MA) has been increasing enormously compared with primary studies [1]. Rigorously conducted SRs are believed to provide the most reliable information in healthcare and are often fundamental to developing practice guidelines [2]. However, recently, the rigorousness of numerous studies published as SRs and MAs has been questioned due to methodological limitations and lack of adherence to international standards [3]. A number of studies in various fields of medicine and health sciences have addressed this issue [4,5,6,7,8], confirming important shortcomings [9]. 

Several tools have been developed to guide the methodological and reporting quality of SR/MAs [10,11]. However, the use of these tools and the quality of studies published as SR/MAs is less than optimal [9,12]. One of the commonly used and widely accepted instrument is AMSTAR-2 (Assessing the Methodological Quality of Systematic Reviews) [13]. The current version is an update of the original AMSTAR instrument published in 2007, which was dedicated to reviews of randomized controlled trials (RCTs) [13]. The AMSTAR-2 version allows for an assessment of reviews including both randomized and nonrandomized studies [14]. Another widely used instrument, ROBIS (Risk Of Bias In Systematic reviews), was designed for assessing the risk of bias (RoB) in all types of SRs of randomized trials and nonrandomized studies [15]. It helps identify concerns within the review process regarding the identification, selection, and appraisal of studies and the collection and synthesis of data, as well as the formulation of conclusions. 

Previous studies explored various predictors of the methodological and reporting quality of SRs in various fields of medicine and health sciences. However, they did not specifically address studies on nutrition in cancer prevention [16]. Therefore, within the context of a project addressing the quality and risk of bias of studies published as SR/MAs on nutrition in cancer prevention, we aimed to identify predictors of higher quality (defined using AMSTAR-2 instrument) or lower RoB (defined using the ROBIS instrument) among SR/MAs.

## 2. Materials and Methods

### 2.1. Search and Selection of Studies

The methods for this systematic survey are described in detail in our previous paper [17]. In brief, following pre-specified protocol (PROSPERO CRD42019121116), we searched three databases (MEDLINE, Embase, Cochrane Library) for studies published as systematic reviews/meta-analyses (SR/MA) on nutritional interventions/exposures for cancer prevention using keywords related to cancer (e.g., ‘oncology’/, ‘neoplasm’/, or ‘leukemia’/), nutritional intervention/exposure (e.g., ‘dairy product’, ‘milk’, or ‘fish product’/), and systematic review or meta-analysis (e.g., ‘systematic review’, ‘meta-analysis’). The last search was carried out on 3 November 2018. Detailed search strategy and inclusion criteria were reported previously [17]; in brief, we were interested in articles identified as SRs/MAs, published from 2010 onward, which investigated, on the basis of included primary comparative studies, the effects of nutritional or dietary interventions or exposures on cancer (any cancer incidence or any cancer mortality) in the general population or people at risk of cancer.

Of the 24,739 references from SR/MAs published between 2010 and 2018, after removing duplicates, we screened 20,413 records, which yielded 1586 full texts for further analysis. We identified 737 SR/MAs reported in 746 records (see Figure S1). From this sample, using the RAND procedure in Microsoft Excel, we randomly selected 101 studies. The number of articles selected per year was proportional to the number of eligible SR/MAs published in the same year. Following piloting extraction forms and calibration exercises that involved all participating reviewers, pairs of two reviewers independently extracted the data and assessed both the quality and RoB. 

### 2.2. Assessment of the Quality and Risk of Bias of Included SR/MA

To obtain the quality/RoB assessments for each of the included studies, we used AMSTAR-2 and ROBIS, according to the published guidance documents [14,15]. The AMSTAR-2 contains a total of 16 items. The developers of the AMSTAR-2 tool suggested seven of the items be more important (critical domains), which include items related to protocol content and registration (item 2), comprehensive research searches (item 4), providing reasons for exclusion of research (item 7), adequate evaluation of study quality (item 9) and its influence on the results (item 13), proper synthesis of results (item 11), and investigation of the presence/impact of publication bias/small study effect (item 15). Among the remaining items, nine non-critical domains are assessed. They include the research question (item 1), explanation for study design selection (item 3), the transparency of the studies identification and extraction process (item 5 and 6), adequate characteristics of included papers (item 8), including funding (item 10), the impact of quality on the synthesized results (item 12), explaining heterogeneity (item 14), and reporting conflicts of interest and financing (item 16). The overall summary assessment took into account the number of critical domains that received a "No". The studies were judged to be of high quality if the included reviews did not contain any major flaws in critical domains; of moderate quality if there were no flaws in the critical domains, but more than one flaw was identified in the non-critical domains; low quality if it had one major flaw in a critical domain; and critically low quality if it had more than one major flaw in a critical domain.

In the current analysis for the AMSTAR-2 instrument, the number of studies assessed as ‘high quality’ was too small to conduct meaningful analyses using only the overall final assessment. Therefore, we recorded the number of ‘No’ answers for items identified as critical and those identified as noncritical by the instrument developers, as well as the total number of ‘No’ answers for all items for each study in the full AMSTAR-2 assessment. Since critical items 11 (proper synthesis of results) and 15 (presence of the publication bias/small study effect) of the AMSTAR-2 tool do not apply to studies without MA, we conducted two separate analyses: one for all studies without those two items and the other for studies that included MA for all AMSTAR-2 items. In case of items that included two parts (item 9 for RCT and non-RCT (adequate evaluation of study quality) and item 11 for RCT and non-RCT), the final answer was the lowest of the two parts (if the answer was ‘No’ in any of the parts, it was classified as ‘No’ for the whole domain).

The ROBIS tool was explicitly designed to assess the RoB in SRs. The assessment is carried out in three steps. These include (1) relevance assessment (optional); (2) identifying concerns connected with the review process; and (3) judgement of the risk of bias. The review process is assessed in four domains that may introduce bias: (1) study eligibility criteria (5 questions); (2) identification and selection of studies (5 questions); (3) data collection and study appraisal (5 questions); and (4) synthesis and findings (6 questions). Each domain may be judged as having low, unclear, or high levels of concern. Similar levels apply to the entire review’s risk of bias assessment, where review findings’ interpretation and limitations are considered [15]. Overall risk of bias in the review can be assessed as low, high, or unclear.

In the current analysis for the ROBIS tool, the number of studies assessed as having low RoB was too small to perform meaningful analyses using only the final overall assessment. Therefore, we used two approaches. In approach 1, we calculated the total number of items within the instrument for which the responses were ‘Yes’ or ‘Probably Yes’, without taking into account the domains (total number of possible ‘Yes’ responses was 21). In approach 2, we considered the structure of the instrument (four domains) and analyzed each study by the number of domains for which it was assessed as ‘low concern for bias’.

### 2.3. Characteristics Analyzed as Predictors

For each included study, we extracted the data on the following characteristics (also shown in Table S1): form of intervention/exposure (supplement (vitamin/dietary/mineral)/other); inclusion of RCTs (no/yes); publication year (analyzed in the following categories: 2010–2012, 2013–2015, and 2016–2018); country of the corresponding author (authors from China/outside of China); impact factor value; reported information about the protocol (no/yes); search date > 12 months before publication (no/yes/not reported); reported search for unpublished studies (no/yes/not reported); reported use of any quality/RoB assessment for primary studies included (no/yes); reporting any subgroups (no/yes); use of tests of interaction for subgroup analyses (no/yes/not applicable); reported any sensitivity analysis (no/yes); reported publication bias assessment (no/yes); reported use of GRADE (Grading of Recommendations, Assessment, Development and Evaluations) or other methods for assessing overall certainty of evidence (no/yes); referring to the use of PRISMA or MOOSE statement (no/yes); and referring to use of the Cochrane Handbook or other methodological guidance, e.g., Agency for Healthcare Research and Quality, Joanna Briggs Institute (no/yes). 

### 2.4. Data Analysis 

We explored whether any of the aforementioned characteristics (the list of characteristics explored as predictors was consistent with the literature [5,6,18,19,20]) were associated with the higher quality of SR/MAs operationalized using a number of "No" responses (the higher number, the lower quality) on full AMSTAR-2 assessment and "Yes"/"Probably Yes" responses (the higher number, the higher quality/lower concern for risk of bias) on full ROBIS assessment. 

We examined the associations for each of the specified characteristics with each AMSTAR-2 item, as well as the number of ‘No’ answers in items indicated as critical, noncritical, and all AMSTAR-2 items. 

For the ROBIS tool, we examined the association of the specified characteristics and assessments in each of the ROBIS domains (high/low/unclear in four domains of the tool) and the total number of ‘Yes’/’Probably Yes’ answers for all items. 

Frequencies were compared using the χ^2^ or Fisher’s exact test. We used the Poisson regression model to identify the predictors of the number of ‘No’ answers in AMSTAR-2 for critical (2, 4, 7, 9, 11, 13, 15), noncritical (1, 3, 5, 6, 8, 10, 12, 14, 16), and all items. In the ROBIS analysis using approach 1, we used the Poisson regression model for the predictors of the number of ‘Yes’ or ‘Probably Yes’ answers for all ROBIS items. For approach 2 (domain-specific analysis using the ROBIS tool), we dichotomized the studies into those that had at least one domain assessed as having low concern for bias and all other studies. We used logistic regression for the analysis. First, we performed the univariate model for each variable. Subsequently, we repeated the analyses using the multivariable model including all variables with a p value of less than 0.1 in the univariate analysis. The results of Poisson regression are presented as an incidence rate ratio (IRR) with 95% confidence interval (95% CI). The results of logistic regression are presented as odds ratio (OR) with 95% CI. All statistical analyses were performed using the IBM SPSS Statistics for Windows software (Version 26.0, released 2019; IBM Corp., Armonk, NY, USA).

## 3. Results

### 3.1. Characteristics of Included Studies

The analysis included 101 studies randomly selected from 737 studies meeting the inclusion criteria. The studies were described in detail previously [17]. Briefly, the included studies focused on a specific type of food (35%, e.g., dairy) or nutrients (27%, e.g., polyphenols) or nonalcoholic drink (16%, e.g., tea). Studies investigated the incidence of a single type of cancer (73%), multiple types of cancer (21%), or any cancer (6%). Investigated publications included various study designs, such as cohort studies (93%), case-control studies (81%), and RCTs (21%).

Summary information on the distribution of factors analyzed as predictors are presented in Supplementary Materials, Table S1.

### 3.2. Quality of Included Studies 

A detailed description of the specific items of both tools that were most frequently met or not met can be found in the previous publication [17]. Briefly, 98 of the 101 included studies were assessed as having critically low quality based on AMSTAR-2 assessment, defined as the presence of more than one critical flaw with or without noncritical weaknesses. Only one study was assessed as ‘high quality’ based on the seven critical items, while two studies had one concern among the critical items, indicating low quality. As for ROBIS, only 3 of the included studies (3%) were assessed as having low RoB, while 98 (97%) were assessed as having high overall RoB. The least well-respected AMSTAR-2 critical item was item 2—protocol and its content—while for ROBIS it was item 2.1 (appropriate range of sources searched). Furthermore, none of the critical items for AMSTAR-2 were fully met by at least 50% of studies, while vast majority of studies adequately reported authors’ conflict of interest and sources of funding (AMSTAR-2 item 16) and collected relevant study results for synthesis (item 3.3 of ROBIS).

### 3.3. Predictors of Methodological Quality

The identified predictors of the number of ‘No’ answers in AMSTAR-2 considered in at least one multivariable model are presented in Table 1. Variables analyzed only in the univariate models are presented in Supplementary Materials, Table S2.

In the analysis of AMSTAR-2 critical domains in all included studies, the use of at least one of the quality or RoB assessment tools (e.g., Cochrane Risk of Bias tool, Newcastle–Ottawa scale) was associated with a significantly lower number of ‘No’ answers both in univariate and multivariable analyses. Furthermore, information about the protocol for the review was associated with a nonsignificantly lower number of ‘No’ answers in univariate analysis and a nonsignificant trend in multivariable analysis. 

In our analysis among eligible SRs including MA, both of these characteristics (i.e., information about the protocol for the review and use of at least one quality or RoB assessment tool) were associated with a significantly lower number of ‘No’ answers both in univariate and multivariable analyses. 

For noncritical domains, the only characteristic that showed a significant association in the analysis of SRs with MA in both univariate and multivariable analysis was the use of at least one quality or RoB assessment tool, while referring to use of the Cochrane Handbook or other methodological guidelines showed significant association in univariate analysis and nonsignificant trend in multivariable analysis. However, in the analysis of all studies, the use of at least one quality or RoB assessment tool was associated only with a trend in the multivariable analysis, while referring to use of the Cochrane Handbook or other methodological guidelines showed significant association in both univariate and multivariable analysis. 

In the analysis of all AMSTAR-2 domains, information about the protocol for the review, information about the search for unpublished studies, and the use of at least one quality or RoB assessment tool were associated with a lower number of ‘No’ answers in all studies in univariate and multivariable analyses. On the other hand, in the analysis of studies that performed MA, a significant association in both univariate and multivariable analyses was found for the use of at least one quality or RoB assessment tool and referring to use of the Cochrane Handbook or other methodological guidelines, while a trend was observed for information about the protocol. 

Variables that showed some associations in univariate models included the use of GRADE or other methods for assessing the overall certainty of evidence, inclusion of RCTs and the year of publication (especially 2016–2018 vs. 2010–2012).

### 3.4. Predictors of the Risk of Bias

The identified predictors of the number of ‘Yes’ or ‘Probably Yes’ answers in the ROBIS instrument taking into account at least one multivariable model are presented in Table 2. Variables analyzed only in the univariate models are presented in Supplementary Materials, Table S3.

Similar to the results for the AMSTAR-2 tool, we identified the following characteristics to be associated with a higher number of ‘Yes’ or ‘Probably Yes’ answers for all ROBIS items in both univariate and multivariable analyses: information about the protocol for the SR/MA, information about the search for unpublished studies, the use of at least one quality or RoB assessment tool, and the country of the corresponding author.

In the analysis of at least one domain assessed as having low concern for bias, we observed a significant association for the use of at least one quality or RoB assessment tool in both univariate and multivariable analyses, as well as a trend for information about the protocol and the search for unpublished studies.

The year of publication, search within 12 months, inclusion of RCTs, clearly specified type of outcome, type of intervention/exposure (supplement (vitamin/dietary/mineral)), the use of GRADE or other methods to assess the overall certainty of evidence, and referring to use of the Cochrane Handbook or other methodological guidelines were associated with a higher number of ‘Yes’ or ‘Probably Yes’ answers in the univariate models. On the other hand, the inclusion of RCTs and the use of GRADE or other methods to assess the overall quality of evidence were associated with four- and ten-fold higher odds, respectively, of at least one domain being assessed as ‘low concern for bias’ (Table S3).

In the detailed analysis by the ROBIS domains, we determined the following characteristics to be associated with a significantly higher rate of ‘low concern for bias’ assessment in more than one ROBIS domain: information about the protocol of the review, inclusion of RCTs, information about the search for unpublished studies, use of at least one quality or RoB assessment tool, and the use of GRADE or other methods to assess the overall certainty of evidence. Data are presented in Supplementary Materials, Table S4.

## 4. Discussion

### 4.1. Main Findings

In our analysis of studies published as SRs with or without MA, two variables emerged as significant predictors of the lower number of ‘No’ responses for AMSTAR-2 items and a higher number of ‘Yes’ or ‘Probably Yes’ responses for ROBIS items in the majority of multivariable analyses. These included information about a protocol and the use of at least one quality/RoB assessment tool for individual studies included in the SR/MA. Referring to use of methodological guidelines and information about the search for unpublished studies/data were also associated with a lower number of ‘No’ responses in AMSTAR-2 and a higher number of ‘Yes’ or ‘Probably Yes’ responses in ROBIS. Moreover, not using at least one RoB assessment tool to assess the quality/RoB of individual studies included in the SR/MA was associated with lower odds that a study would have a ‘low concern for bias’ assessment in at least one domain of the ROBIS tool.

### 4.2. Previous Studies

In line with our study, research in nutrition and other fields of medicine and health sciences indicated the lack of a protocol as an item associated with lower methodological or reporting quality of SR/Mas [12,21,22,23,24,25]. Furthermore, there is evidence that the quality of reviews has improved since the launch of SR/MA protocol registration (e.g., PROSPERO) [26]. In a survey of global researchers, Tawfik et al. [27] showed that almost half of the authors of reviews do not register protocols a priori, most often due to the lack of knowledge. 

Despite the fact that several previous studies reported insufficient assessment of RoB of primary studies included in SR/MAs [21,28,29,30,31], we did not identify any studies that would formally test such a variable as a predictor of SR quality. However, previous studies showed that involvement of a ‘research methodologist’ predicts a higher quality of SRs. This might partially explain our findings because investigators with expertise in SR/MA methodology presumably make the SR/MA team aware of the need to critically assess RoB among the included studies and to use the assessments when interpreting SR/MA outcomes [32].

As for the year of publication, the authors of previous studies indicated that inclusion of more recent publications was associated with a higher quality of SR/MAs, as compared with older publications. This is in line with the results of our univariate analyses [4,18,21,24,25,28,30,33]. 

Several previous studies reported that the journal’s impact factor was a significant predictor of higher methodological or reporting quality of SR/MAs [24,30,32,34]. However, this is in contrast to the results of our univariate analyses. In our sample, similar to the study by Remschmidt et al. [35], the impact factor value was not associated with higher quality SR/MAs [24,30,34]. These discrepancies might be explained by the fact that samples were derived from different fields of medicine and health sciences, with a different distribution of impact factor values. Another possible explanation is the fact that, unlike medicine, nutrition science has not embraced the need for high-quality SR/MAs to inform practice and policy [12], as evidenced in part by the poor quality of dietary guidelines throughout the field [36,37,38,39]. 

Previous studies also indicated that the research institution or the origin of the corresponding author could also predict a higher quality of the SR/MA [22,28,30,32,33,40,41,42,43]. However, in our sample, a lower RoB (higher number of ‘Yes’ or ‘Probably Yes’ answers) was characteristic of reviews published by investigators from China only in the ROBIS assessment. On the other hand, Xu et al. [33,40] reported a higher quality of European reviews when compared with American [33] and Asian-Pacific ones [40], while Tian et al. [44] concluded that there were no differences between the quality of reviews published by authors from China and the United States.

Previous studies also revealed other predictors of SR/MA quality, including the number of authors [21,30,33,40,43,45,46], inclusion of RCTs only [41], or the use of language restrictions [34]. However, this was not confirmed by our study. This discrepancy could be explained by the different characteristics of the included reviews (e.g., predominantly based on RCTs), the analysis of specific SR types only (such as dose–response meta-analyses), or characteristics that may differ between the various fields of medicine.

To our knowledge, this is the first study to assess predictors of the quality of SRs addressing nutrition in cancer prevention. Previous studies in this field focused on the assessment of methodological or reporting quality of SR/MAs in general, without evaluating potential predictors of higher quality [12,28,46].

### 4.3. Strengths and Limitations

The strength of our study lies in adherence to the rigid methodology of performing SRs based on standard recommended methods, along with the development of a study protocol for our survey [47]. In addition to providing credibility through the work of pairs of two independent reviewers, each step was preceded by calibration between screeners, data extractors, and quality assessors. We also considered different years of publication, demonstrating that the quality improved over time, and the 101 SR/MAs included in our analysis were selected randomly, with a distribution proportional to the number of publications per year. 

Our review also has a number of limitations. First, this study was a part of the project assessing characteristics and quality of studies published as systematic reviews, which was commenced in 2018. Therefore, we arbitrarily searched for SR/MAs published over nine years, and it is possible that the quality of SR/MAs in the field has improved in recent years. However, a 2021 systematic survey of the quality of reviews in the general field of nutrition has suggested that reviews have not tended to improve [12]. Therefore, we assume that an updated search would not affect our findings. Second, the overall quality of eligible SR/MAs based on summary scores from AMSTAR-2 and ROBIS was poor (i.e., only 3 of the 101 reviews using AMSTAR-2 were not judged to be of critically low quality, while 3 of the 101 reviews using ROBIS were judged to have low RoB). For this reason, we were unable to use summary scores in our modelling. Instead, we used individual items from each instrument post hoc. Another limitation of our methods includes the discrepancy between the publication of ROBIS (2016) and AMSTAR-2 (2017) and the time period for which SR/MAs were eligible for our study (2010 to 2018). It may be unreasonable to expect that SR/MAs met the quality criteria for ROBIS and AMSTAR-2, given that most authors of our eligible SR/MAs were likely to be unaware of these instruments. Moreover, while it would be interesting to explore whether the quality of SR/MAs improved after the publication of ROBIS and AMSTAR-2, it is unreasonable to expect that these instruments would impact quality in such a short timeframe. Among SR/MAs published in 2018, arguably many would have been submitted prior to AMSTAR-2 (2017) having been published. However, both tools refer to the elements that are vital for systematic review quality/risk of bias, e.g., formulating a research question, comprehensive literature search, or credibility assessment, and both tools refer to elements that are defining systematic reviews and that were present in previously available tools (such as AMSTAR). However, we found that providing information about the study protocol, using any quality/RoB assessment, and search for unpublished studies to be the key predictors of quality, and these findings correspond with the critical AMSTAR-2 items. Finally, among the 16 items of AMSTAR-2 and 21 items of ROBIS, the vast majority of studies (97%) scored poorly. This suggests that SR/MAs in the field of nutrition in cancer prevention are consistently of low quality or have high RoB. It is possible that our conclusions on the predictors of higher-quality reviews would be different if the scores were more evenly distributed.

## 5. Conclusions

To our knowledge, our study was the first attempt to determine the predictors of the quality and RoB of SR/MAs addressing nutrition in cancer prevention. Based on our sample of SRs addressing nutrition in cancer prevention, we demonstrated that the use of quality/RoB assessments and providing information about the SR/MA protocol, followed by referring to use of methodological guidelines and searching for unpublished studies were associated with higher overall adherence to methodological criteria specified in the AMSTAR-2 and ROBIS instruments. Our results have important implications for future conduct and publication of SRs. Authors should closely follow guidance on methodological reporting, while editors and readers should pay particular attention to whether there was a prespecified SR/MA study protocol, whether quality/RoB tools were used to assess all studies included in the SR/MA, and whether a comprehensive literature search was conducted, as these are factors that might indicate a higher quality of SR/MAs.

## Figures and Tables

**Table 1:** Predictors of the number of ‘No’ answers in AMSTAR-2 (Poisson regression model).

Variable (Reference)	Variable	No. of ‘No’ Responses in Critical Domains &&				No. of ‘No’ Responses in Noncritical Domains &&				No. of ‘No’ Responses in All Domains			
		Univariate IRR (95% CI)		Multivariable IRR (95% CI)		Univariate IRR (95% CI)		Multivariable IRR (95% CI)		Univariate IRR (95% CI)		Multivariable IRR (95% CI)	
		All Studies	With MA only	All Studies ^	With MA only ^	All Studies	With MA only	All Studies *	With MA only *	All Studies	With MA only	All Studies #	With MA only &
Information about the protocol of the review (‘No’)	yes	0.66 (0.43–1.02)	0.66 (0.46–0.97)	0.68 (0.44–1.04)	0.67 (0.46–0.98)	0.78 (0.55–1.1)	0.83 (0.60–1.13)	–	–	0.73 (0.56–0.95)	0.75 (0.59–0.96)	0.73 (0.56–0.97)	0.8 (0.62–1.002)
*p* value		0.06	**0.03**	0.07	**0.039**	0.15	0.24	–	–	**0.02**	**0.02**	**0.03**	0.07
Information about the search for unpublished studies/data (‘No’ or NR)	yes	0.64 (0.44–0.92)	0.69 (0.51–0.94)	–	–	0.74 (0.55–0.99)	0.77 (0.58–1.01)	–	–	0.70 (0.55–0.88)	0.73 (0.59–0.90)	0.75 (0.59–0.95)	–
*p* value		**0.02**	**0.02**	–	–	**0.05**	0.06	–	–	**0.002**	**0.003**	**0.02**	–
Use of any quality or RoB assessment tool (at least one tool)	none	1.56 (1.27–1.93)	1.46 (1.21–1.76)	1.56 (1.26–1.92)	1.45 (1.21–1.75)	1.21 (1.02–1.45)	1.32 (1.11–1.56)	1.18 (0.98–1.41)	1.29 (1.08–1.3)	1.35 (1.18–1.54)	1.38 (1.22–1.57)	1.26 (1.09–1.45)	1.35 (1.18–1.53)
*p* value		**<0.001**	**<0.001**	**<0.001**	**<0.001**	**0.03**	**0.002**	0.07	**0.004**	**<0.001**	**<0.001**	**0.001**	**<0.001**
Country of the corresponding author (outside of China)	China	0.89 (0.73– 1.1)	0.93 (0.78–1.12)	–	–	0.88 (0.74–1.06)	0.87 (0.74–1.04)	–	–	0.89 (0.78–1.02)	0.90 (0.8–1.02)	0.87 (0.76–1.01)	–
*p* value		0.29	0.47	–	–	0.17	0.12	–	–	0.08	0.1	0.07	–
Referring to use of the Cochrane Handbook or other methodological guidelines (‘None’)	At least 1	0.71 (0.51–0.99)	0.70 (0.53–0.94)	-	-	0.72 (0.54–0.95)	0.75 (0.58–0.97)	0.75 (0.56–0.99)	0.8 (0.61–1.04)	0.72 (0.58–0.89)	0.73 (0.6–0.88)	-	0.81 (0.67–0.99)
*p* value		**0.045**	**0.02**	-	-	**0.02**	**0.03**	**0.04**	0.09	**0.0002**	**0.001**	-	**0.04**

A *p* value was less than 0.1 in the univariate analysis for all listed variables that were taken into account in at least one multivariable model. Statistically significant results are bolded. ^ Included in the model: information about the protocol, use of quality/RoB tool; multivariable adjusted for the variables included. * Included in the model: use of quality/RoB tool, referring to the use of the Cochrane Handbook or other methodological guideline; multivariable adjusted for the variables included. # Included in the model: information about the protocol, information about the search for unpublished data, use of quality/RoB tool, country of the corresponding author; multivariable adjusted for the variables included. & Included in the model: information about the protocol, use of quality/RoB tool, referring to the use of the Cochrane Handbook or other methodological guideline; multivariable adjusted for the variables included. && The critical domains include: protocol content and registration (item 2), comprehensive research searches (item 4), argumentation for exclusion of research (item 7), adequate evaluation of study quality (item 9) and its influence on the results (item 13), proper synthesis of results (item 11), and investigation of the presence/impact of the publication bias/small study effect (item 15). The non-critical domains include: the research question (item 1), explanation for study design selection (item 3), the transparency of the studies identification and extraction process (item 5 and 6), adequate characteristics of included papers (item 8), including funding (item 10), the impact of quality on the synthesized results (item 12), explaining heterogeneity (item 14), and reporting conflicts of interest and financing (item 16). IRR = incidence rate ratio; NR = not reported; NA = not applicable; MA = meta-analysis; RoB = risk of bias; RCT = randomized controlled trial.

**Table 2:** Predictors of the number of ‘Yes’ or ‘Probably Yes’ responses in ROBIS (Poisson or logistic regression model) and at least one of the ROBIS domains assessed as having low concern for bias (logistic regression).

Variable (Reference)	Variable	No. of ‘Yes’ or ‘Probably Yes’ Responses in ROBIS (Poisson Regression Model) #		At Least One Domain Assessed as Having ‘Low Concern for Bias’ (Logistic Regression Model)	
		Univariate IRR (95% CI)	Multivariable IRR (95% CI) *	Univariate OR (95% CI)	Multivariable OR (95% CI) ^
Information about the protocol of the review (‘No’)	**yes**	1.46 (1.21–1.76)	1.43 (1.17–1.74)	5.89 (1.37– 25.31)	6.33 (0.87–46.2)
*p* value		**<0.001**	**<0.001**	**0.017**	0.06
Information about the search for unpublished studies/data (‘No’ or NR)	yes	1.42 (1.21–1.67)	1.28 (1.07–1.52)	7.524 (2.071–27.336)	4.191 (0.932– 18.852)
*p* value		**<0.001**	**0.007**	**0.002**	0.06
Use of any quality or RoB assessment tool (at least one tool)	none	0.70 (0.62–0.80)	0.76 (0.66–0.87)	0.057 (0.007–0.449)	0.061 (0.007–0.527)
*p* value		**<0.001**	**<0.001**	**0.007**	**0.01**
Country of the corresponding author (outside of China)	China	1.12 (0.99–1.26)	1.15 (1.006–1.31)	0.477 (0.151–1.513)	–
*p* value		0.08	**0.04**	0.21	–

A *p* value was less than 0.1 in the univariate analysis for all listed variables that were taken into account in at least one multivariable model. Statistically significant results are bolded. * Included in the model: information about the protocol, search for unpublished data, use of quality/RoB tool, country of the corresponding author; multivariable adjusted for the variables included. ^ Included in the model: information about the protocol, search for unpublished data, use of quality/RoB tool; multivariable adjusted for the variables included. # The assessment was carried out in four domains that may introduce bias: (1) study eligibility criteria (5 questions); (2) identification and selection of studies (5 questions); (3) data collection and study appraisal (5 questions); and (4) synthesis and findings (6 questions). NR = not reported; NA = not applicable; MA = meta-analysis; OR = odds ratio; RoB = risk of bias.

## Data Availability

The datasets used and/or analyzed during the current study are available from the corresponding author on reasonable request.

## Supplementary Materials

The following are available online at https://www.mdpi.com/article/10.3390/ijerph19010506/s1, Table S1: Summary information on the factors analyzed as predictors based on all studies (101 studies), Table S2: Predictors of the number of ‘No’ answers in AMSTAR-2 (Poisson regression model). Listed variables were only used in univariate analyses and were not included in any of multivariable models (*p* value in all analyses > 0.1), Table S3: Predictors of the number of ‘Yes’ or ‘Probably Yes’ responses in ROBIS (Poisson regression) and at least one domain assessed as low risk of bias (logistic regression model), Table S4: Detailed analysis by ROBIS domain, Figure S1: Study flowchart.
