# Supplementary material for: Predictors of Higher Quality of Systematic Reviews Addressing Nutrition and Cancer Prevention
Source: Int J Environ Res Public Health. 2022 Jan 3;19(1):506. doi: 10.3390/ijerph19010506 (PMC8744691; doi:10.3390/ijerph19010506)

Table S1. Summary information on the factors analysed as predictors based on all studies (101 studies)		
**Variable**	**Variable**	**% (n)**
Elements of PICOT question		
Category (form) of intervention/exposure	Supplement (vitamin/dietary/mineral)	8.9 (9)
	Others	91.1 (92)
Number of outcomes analysed in SR/MA	Continuous	1 (1-23)*
Type of outcomes studied	Unclear	10.9 (11)
	Clearly specified	89.1 (90)
Type of outcomes studied (n=90)	Only incidence or only mortality	75.2 (76)
	Both	13.9 (14)
Included RCTs	Yes	13.9 (14)
	No	86.1 (87)
General information		
Total number of included studies in SR/MA	Continuous	18 (5-572)*
Year of publication	2010-2012	21.8 (22)
	2013-2015	41.6 (42)
	2016-2018	36.6 (37)
Country of the corresponding author	China	48.5 (49)
	Other than China	51.5 (52)
	No	35.6 (36)
Impact factor value	Continuous	3.53 (0–51.27)*
Number of authors	Continuous	6 (1–48)*
Systematic review process		
Information about the protocol of the review	Yes	8.9 (9)
	No	91.1 (92)
Over 12 months between search and publication date	Yes	25.7 (26)
	No	70.3 (71)
	NR	4.0 (4)
Information about the search for unpublished studies/data	Yes	12.9 (13)
	No	87.1 (88)
Use of any applicable quality/RoB assessment for primary studies	Yes	51.5 (52)
	No	48.5 (49)
Systematic review analysis		
Reporting any subgroups	Yes	80.2 (81)
	No	19.8 (20)
Use of tests of interaction for subgroup analyses	Yes	13.9 (14)
	No	66.3 (67)
	Not applicable	19.8 (20)
Publication bias/small study analysis	Yes	82.2 (83)
	No	17.8 (18)
Was any sensitivity analysis reported	Yes	65.3 (66)
	No	34.7 (35)
Total number of meta-analyses reported	Continuous	3 (0–64)*
Referring to methods		
Was the use of GRADE or other tool for overall quality-of-evidence assessment reported	Yes	5.0 (5)
	No	95.0 (95)
Referring to the use of PRISMA or MOOSE for reporting	One	42.6 (43)
	Both	4.0 (4)
	None	53.5 (54)
Referring to use of the Cochrane Handbook or other methodological guidelines	Yes	13.9 (14)
	Both	1.0 (1)
	None	85.1 (86)
MA – metaanalysis; RCT - randomized controlled trials; RoB – risk of bias; SR – systematic review<br>*median (range)		

Table S2. Predictors of the number of 'No' answers in AMSTAR-2 (Poisson regression model). Listed variables were only used in univariate analyses and were not included in any of multivariable models (p value in all analyses >0.1)							
**Variable (reference)**	**Variable**	**No. of 'No' responses in critical domains* Univariate IRR (95% CI)**		**No. of 'No' responses in non-critical domains* Univariate IRR (95% CI)**		**No. of 'No' responses in all domains Univariate IRR (95% CI)**	
		**All studies**	**With MA only**	**All studies**	**With MA only**	**All studies**	**With MA only**
Category (form) of intervention /exposure (other)	Supplement (vitamin/dietary/mineral)	0.89 (0.77–1.64)	0.87 (0.58–1.31)	0.83 (0.59–1.15)	0.77 (0.55–1.09)	0.85 (0.66–1.10)	0.82 (0.64–1.04)
p value		0.59	0.51	0.26	0.14	0.22	0.12
Number of outcomes analyzed		1.01 (0.98–1.03)	1.01(0.99–1.03)	1.00 (0.98–1.03)	1.01 (0.99–1.03)	1.01 (0.99–1.02)	1.01 (0.99–1.02)
p value		0.67	0.3	0.73	0.56	0.59	0.26
Type of outcome studied (only incidence or only mortality)	Both incidence and mortality	1.04 (0.77– 1.40)	1.09 (0.84–1.41)	0.94 (0.72–1.22)	0.96 (0.75–1.23)	0.98 (0.80–1.19)	1.02 (0.85–1.22)
p value		0.79	0.53	0.62	0.75	0.84	0.85

Type of outcomes studied (unclear)	Clearly specified	0.88 (0.64–1.21)	0.83 (0.63 –1.09)	1.02 (0.77–1.36)	0.98 (0.75–1.28)	0.96 (0.77–1.18)	0.91 (0.75–1.10)
p value		0.43	0.18	0.9	0.9	0.68	0.31
Included RCTs ('No')	Yes	0.8 (0.58-1.10)	0.79 (0.59-1.07)	0.81 (0.61-1.07)	0.77 (0.58-1.01)	0.80 (0.65-0.99)	0.78 (0.64-0.95)
p value		0.17	0.12	0.13	0.07	**0.04**	**0.01**
Total number of included studies		1.001 (0.999–1.002)	1.001 (0.999–1.002)	1.00 (1.00–1.00)	1.00 (1.00–1.00)	1 (1–1)	1 (1–1)
p value		0.38	0.33	0.33	0.28	0.19	0.15
Impact factor value		1 (0.98–1.02)	1 (0.98–1.01)	1 (0.98–1.01)	1 (0.98–1.01)	1.00 (0.99–1.01)	1.00 (0.99–1.01)
p value		0.99	0.88	0.5	0.8	0.58	0.77
Year of publication (2010–2012)	2013–2015	0.91 (0.71–1.18)	0.95 (0.75–1.21)	0.91 (0.73–1.14)	0.91 (0.73–1.13)	0.91 (0.77–1.08)	0.93 (0.79–1.09)
p value		0.50	0.68	0.4	0.41	0.28	0.38
Year of publication (2010–2012)	2016–2018	0.75 (0.57–0.99)	0.8 (0.62–1.03)	0.83 (0.65–1.05)	0.83 (0.65–1.04)	0.8 (0.67–0.95)	0.81 (0.69–0.96)

p value		**0.04**	0.08	0.11	0.12	**0.01**	**0.02**
Over 12 months between search and publication date ('No')	NR	1.45 (0.92–2.29)	1.42 (0.90–2.23)	1.19 (0.78–1.82)	1.16 (0.73–1.84)	1.3 (0.95–1.77)	1.28 (0.93–1.77)
p value		0.11	0.7	0.42	0.52	0.11	0.13
Over 12 months between search and publication date ('No')	Yes	1.07 (0.85–1.36)	0.96 (0.77–1.19)	1.03 (0.84–1.26)	1.06 (0.87–1.28)	1.05 (0.9–1.22)	1.01 (0.87–1.17)
p value		0.57	0.12	0.8	0.59	0.58	0.89
Reporting any subgroups (no)	Yes	0.95 (0.72–1.23)	0.97 (0.72–1.31)	0.92 (0.73–1.15)	0.93 (0.74–1.18)	0.93 (0.78–1.11)	0.93 (0.78–1.11)
P value		0.68	0.85	0.47	0.57	0.41	0.43
Was any sensitivity analysis reported ('No')	Yes	0.91 (0.73–1.13)	0.92 (0.76–1.13)	0.85 (0.71–1.02)	0.86 (0.72–1.03)	0.88 (0.76–1.01)	0.89 (0.78–1.02)
p value		0.38	0.43	0.08	0.11	0.06	0.08
Publication bias analysis (no)	Yes	0.95 (0.73–1.24)	0.95 (0.73–1.24)	0.96 (0.77–1.21)	1.00 (0.78–1.27)	0.96 (0.81–1.14)	0.96 (0.81–1.14)
p value		0.74	0.71	0.74	0.97	0.63	0.63
Use of interaction tests (no)	Yes	–	–	–	0.91 (0.71–1.17)	–	0.96 (0.80–1.15)

p value					0.48		0.66
Use of interaction test (no)	NA	–	–	–	1.20 (0.87–1.66)	–	1.19 (0.94–1.52)
p value					0.27		0.15
Total number of meta-analyses reported		1.01 (0.99–1.02)	1.00 (0.99–1.01)	1.00 (0.99–1.01)	1.00 (0.99–1.01)	1.00 (1.00–1.01)	1.00 1.00–1.01)
p value		0.39	0.43	0.76	0.53	0.43	0.32
Was the use of GRADE or other tool for overall quality-of-evidence assessment reported ('No')	Yes	0.38 (0.18–0.81)	0.46 (0.25–0.83)	0.64 (0.39–1.06)	0.68 (0.43–1.07)	0.53 (0.35–0.81)	0.57 (0.40–0.83)
p value		**0.01**	**0.01**	0.08	0.11	**0.003**	**0.003**
Referring to the use of PRISMA or MOOSE for reporting ('None')	At least 1	0.915 (0.74-1.13)	0.96 (0.8-1.16)	0.96 (0.8-1.14)	0.96 (0.81-1.14)	0.94 (0.82-1.08)	0.96 (0.85-1.09)
p value		0.4	0.67	0.63	0.67	0.38	0.55

Statistically significant results are bolded.

IRR = incidence rate ratio; NR = not reported; NA = not applicable; MA = meta-analysis; RoB = risk of bias

*The critical domains include: protocol content and registration (item 2), comprehensive research searches (item 4), argumentation for exclusion of research (item 7), adequate evaluation of study quality (item 9) and its influence on the results (item 13), proper synthesis of results (item 11) and investigation of the presence/impact of the publication bias/small study effect (item 15).

The non-critical domains include: the research question (item 1), explanation for study design selection (item 3), the transparency of the studies identification and extraction process (item 5 and 6), adequate characteristics of included papers (item 8), including funding (item 10), the impact of quality on the synthesized results (item 12), explaining heterogeneity (item 14) and reporting conflicts of interest and financing (item 16).

**Table S3. Predictors of the number of 'Yes' or 'Probably Yes' responses in ROBIS (Poisson regression) and at least one domain assessed as low risk of bias (logistic regression model).**			
**Variable (reference)**	**Variable**	**No. of 'Yes' or 'Probably Yes' responses in ROBIS# (Poisson regression)**	**At least one domain assessed as 'low risk of bias' (logistic regression)**
		**Univariate IRR (95% CI)**	**Univariate OR (95% CI)**
Category (form) of intervention/exposure (other)	Supplement (vitamin/dietary/mineral)	1.26 (1.03–1.53)	3.333 (0.734–15.133)
p value		**0.02**	0.12
Type of outcomes studied (unclear)	Clearly specified	1.25 (1.01–1.55)	Not applicable*
p value		**0.04**	Not applicable*
Type of outcomes studied (only incidence or mortality)	Both incidence and mortality	1.02 (0.85–1.21)	1.455 (0.352–6.004)
p value		0.87	0.6
Number of outcomes analyzed	–	1.00 (0.98–1.01)	1.03 (0.91–1.17)
p value		0.56	0.65
Were RCTs included ('No')	Yes	1.27 (1.08–1.5)	4.278 (1.194–15.330)
p value		**0.004**	**0.026**
Total number of included studies	–	1.00 (1.00–1.00)	0.988 (0.954–1.023)
p value		0.21	0.49
Year of publication (2010–2012)	2013–2015	1.20 (1.01–1.43)	2 (0.378–10.569)
p value		**0.03**	0.414
Year of publication (2010–2012)	2016–2018	1.28 (1.08–1.52)	1.935 (0.355–10.554)
p value		**0.006**	0.445

Search vs publication date >12 months? ('No')	NR	0.51 (0.33–0.79)	Not applicable*
p value		**0.002**	–
Search vs publication date >12 months? ('No')	Yes	1.03 (0.89–1.18)	Not applicable*
p value		0.71	
Impact factor	–	1.00 (0.99–1.01)	1.002 (0.917–1.095)
p value		0.65	0.96
Was any sensitivity analysis reported ('No')	Yes	1.12 (0.98–1.28)	0.552 (0.182–1.674)
p value		0.11	0.29
Reporting any subgroups (no)	Yes	1.12 (0.95–1.32)	0.870 (0.218–3.466)
p value		0.18	0.84
Publication bias analysis (no)	Yes	1.05 (0.89–1.23)	1.183 (0.297–4.715)
p value		0.6	0.81
Total number of meta-analyses for dietary intake and serum levels reported	–	1.00 (0.99–1.01)	1.006 (0.948–1.067)
p value		0.92	0.95
Was reported use of GRADE or other for overall quality of evidence ('No')	Yes	1.65 (1.32–2.07)	10.375 (1.569–68.595)
p value		**<0.001**	**0.015**
Referring to the use of PRISMA or MOOSE for reporting ('None')	At least 1	1.06 (0.94-1.20)	1.01 (0.34-3.02)
p value		0.33	0.99

Referring to use of the Cochrane Handbook or other methodological guidelines ('None')	At least 1	1.31 (1.12-1.53)	2.48 (0.67-9.17)
p value		**0.001**	0.17

A p value was less than 0.1 in the univariate analysis for all listed variables that were taken into account in at least one multivariable model.

* Impossible to estimate due to insufficient number of studies.

# The assessment is carried out in four domains that may introduce bias: (1) study eligibility criteria (5 questions); (2) identification and selection of studies (5 questions); (3) data collection and study appraisal (5 questions); and (4) synthesis and findings (6 questions).

Statistically significant results are bolded.

NR = not reported; NA = not applicable; MA = meta-analysis; RoB = risk of bias

Table S4. Detailed analysis by ROBIS domain							
	**Information about the protocol of the review**						
	**No (n=92)**			**Yes (n=9)**			
**Domain**	**High**	**Low**	**Unclear**	**High**	**Low**	**Unclear**	**p value***
Concerns eligibility	97.8	1.1	1.1	55.6	44.4	0.0	**<0.001**
Concerns meth. identification	95.7	3.3	1.1	66.7	33.3	0.0	**0.02**
Concerns meth. collection	89.10	9.80	1.10	66.70	22.20	11.10	**0.049**
Concerns meth. synthesis	94.60	2.20	3.30	66.70	22.20	11.10	**0.022**
	**Inclusion of RCTs**						
	**No (n=87)**			**Yes (n=14)**			
	**High**	**Low**	**Unclear**	**High**	**Low**	**Unclear**	
Concerns eligibility	96.6	2.3	1.1	78.6	21.4	0.0	**0.034**
Concerns meth. identification	94.3	4.6	1.1	85.7	14.3	0.0	0.31
Concerns meth. collection	90.8	8.0	1.1	64.3	28.6	7.1	**0.03**
Concerns meth. synthesis	95.4	2.3	2.3	71.4	14.3	14.3	**0.02**
	**Information about the search for unpublished studies**						
	**No or NR (n=88)**			**Yes (n=13)**			
	**High**	**Low**	**Unclear**	**High**	**Low**	**Unclear**	
Concerns eligibility	97.7	1.1	1.1	69.2	30.8	0.0	**0.001**
Concerns meth. identification	96.6	2.3	1.1	69.2	30.8	0.0	**0.002**
Concerns meth. collection	92.0	6.8	1.1	53.8	38.5	7.7	**0.001**
Concerns meth. synthesis	95.5	1.1	3.4	69.2	23.1	7.7	**0.005**
	**Use of GRADE or other methods to assess overall quality of evidence**						
	**No (n=95)**			**Yes (n=5)**			
	**High**	**Low**	**Unclear**	**High**	**Low**	**Unclear**	
Concerns eligibility	96.8	2.1	1.1	40.0	60.0	0.0	**0.01**
Concerns meth. identification	94.7	4.2	1.1	60.0	40.0	0.0	**0.04**

Concerns meth. collection	89.5	9.5	1.1	40.0	40.0	20.0	**0.008**
Concerns meth. synthesis	93.7	2.1	4.2	60.0	40.0	0.0	**0.002**
	**Use of at least one quality or RoB assessment tool**						
	**Yes (n=52)**			**No (n=49)**			
	**High**	**Low**	**Unclear**	**High**	**Low**	**Unclear**	
Concerns eligibility	90.40	9.60	0.00	98.00	0.00	2.00	0.057
Concerns meth. identification	90.40	9.60	0.00	95.90	2.00	2.00	0.21
Concerns meth. collection	75.00	21.20	3.80	100.00	0.00	0.00	**<0.001**
Concerns meth. synthesis	84.60	7.70	7.70	100.00	0.00	0.00	**0.006**

Data are presented as percentage of studies.

Statistically significant results are bolded.

*χ^2 or Fisher test

NR = not reported; NA = not applicable; MA = meta–analysis; meth. = methodological; RoB = risk of bias; RCT = randomized controlled trial

Figure S1. Study flowchart (based on Zajac, J., et al., Are systematic reviews addressing nutrition for cancer prevention trustworthy? A systematic survey of quality and risk of bias **Nutr. Rev. 2021., 10.1093/nutrit/nuab093. In press, modified)**

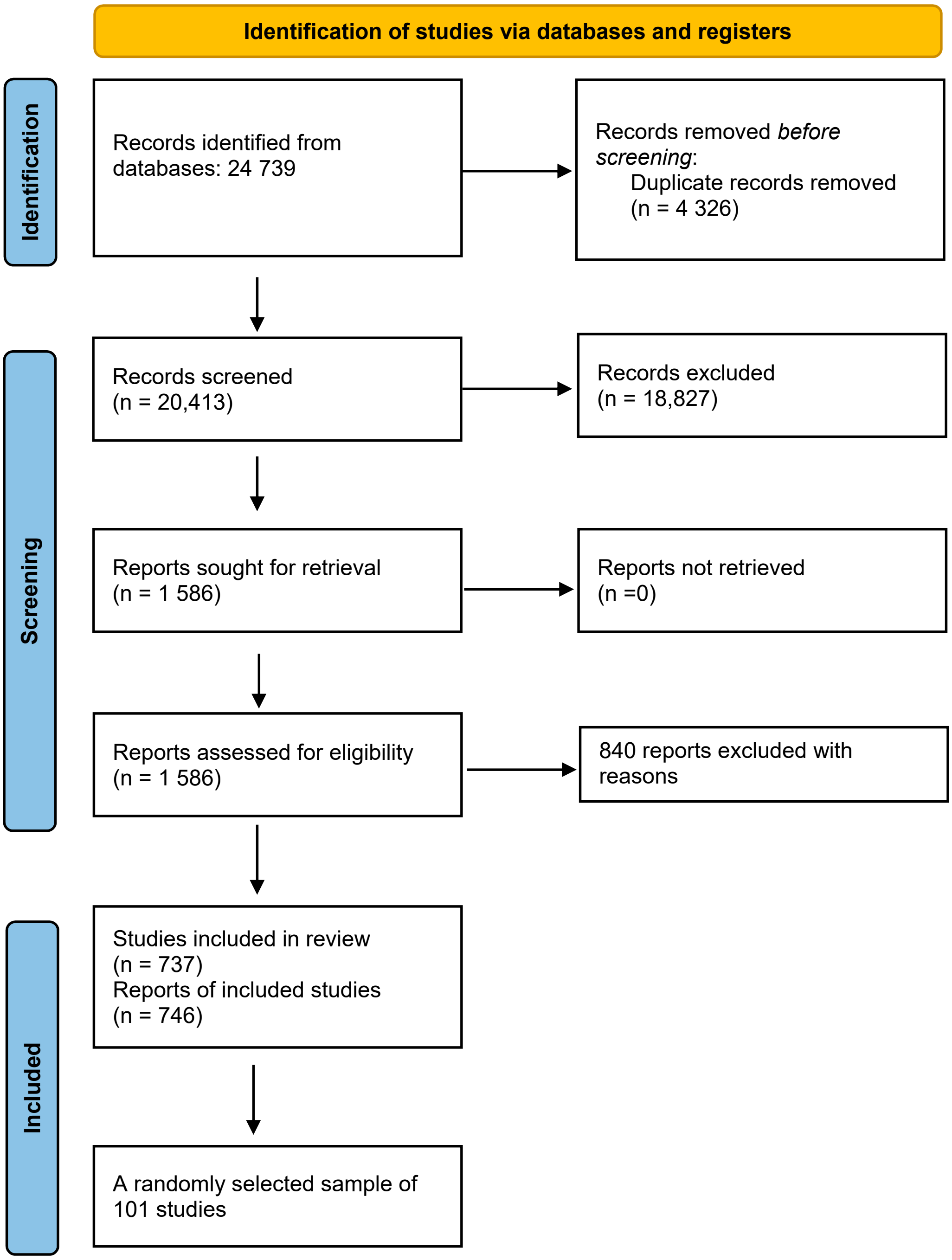

Supplement: Supplementary file 1 [file ijerph-19-00506-s001.zip › ijerph-1447521-supplementary.pdf]
